# Racial identity as a moderator of the association between socioeconomic status and quality of life

**DOI:** 10.3389/fsoc.2022.946653

**Published:** 2022-08-11

**Authors:** Adekunle Adedeji, Johanna Buchcik, Tosin Yinka Akintunde, Erhabor S. Idemudia

**Affiliations:** ^1^Faculty of Humanities, North-West University, Mafikeng, South Africa; ^2^Faculty of Life Sciences, Hamburg University of Applied Sciences, Hamburg, Germany; ^3^Department of Social Work, Chinese University of Hong Kong, Pok Fu Lam, Hong Kong SAR, China

**Keywords:** racial identity, quality of life, socioeconomic status, inequality, South Africa

## Abstract

Research in social and humanitarian science has identified socioeconomic status (SES) as one of the essential determinants of quality of life (QoL). Similarly, racial identity is assumed to predict SES outcomes in multiracial settings. Therefore, understanding how racial identity moderates the association between SES and QoL may provide essential insights into the mechanisms generating socioeconomic inequalities and their implication on life outcomes. The current study employs a cross-sectional study designed to investigate the moderating effect of racial identity on the association between SES and QoL in a sample of 1,049 South Africans. A correlation matrix was computed to explore the bivariate associations between QoL, socioeconomic, and sociodemographic features. ANOVA was used to evaluate racial differences in QoL and SES. A moderator analysis was adopted to determine a possible moderating effect of racial identity on the connection between SES and QoL. Findings show a significant difference in QoL and SES based on race. While racial identity was a significant moderator of the association between QoL and SES for Black Africans, no significant moderating effect was reported for other racial groups. These results highlight the importance of racial identity for life outcomes and emphasis the unique experience associated with Black racial identity and its implications for SES, QoL, and their association in South Africa. This study explains the necessity to improve the QoL of minority groups, such as Black South Africans, and offers detailed explanations of their perceived disadvantage.

## Background

Understanding the determinants of quality of life (QoL) remains crucial for improving individual subjective life evaluation. Research in social and humanitarian science has identified socioeconomic status (SES) as one of the essential determinants of QoL (Rueden et al., 2006; Bielderman et al., 2015). Similarly, more research are focusing on demographic factors, such as racial identity and culture (Kagawa-Singer et al., 2010; Adedeji et al., 2021), as a potential explanator of the linkage between QoL and social indicators.

Quality of life is a multi-dimensional concept relating to the perceived quality of an individual's health, functioning, and daily life (The WHOQOL Group, 1998). While QoL has been established as a crucial indicator for health promotion and disease prevention, the complexity of this concept—encompassing a wide range of dimensions (e.g., social, psychological, physical) and variables (e.g., work and personal relations, positive or negative emotions and behavior, physical pain) (Testa and Simonson, 1996)—makes the QoL susceptible to social, economic, and demographic features and changes. Alongside the continued effort to simplify QoL as an indicator of health and wellbeing, several research projects have identified SES as a determinant for QoL among different populations, groups and in association with other factors (Schoenbaum and Waidmann, 1997; Hanson and Chen, 2007; Bielderman et al., 2015).

Socioeconomic status (SES) is conceptualized as a measure of an individual's combined economic and social status. This construct focuses on three standard measures: income, education, and profession attributed to possible health inequalities and differences in QoL performance (JieAnNaMu et al., 2020). Previous research has commonly argued two avenues through which SES associated with health and wellbeing outcomes: (1) through the ability to purchase or access health-promoting resources and seek treatments (Nikoi and Odimegwu, 2013); and (2) socialization of health habits, for example, health-related lifestyles (smoking, alcohol consumption, diet, and physical activity) (Hanson and Chen, 2007). These avenues are sensitive to demographic features, for example, racial identity.

Several reports have documented different patterns in health behavior and health service utilization among racial minority groups (Egede, 2006; Herbeck et al., 2013). These patterns are often attributed to socioeconomic gaps and experiences of racism, discrimination, or segregation. While the relationship between SES and race is deeply entwined, research has shown that race and ethnicity in terms of stratification often determine a person's socioeconomic status (Carter and Helms, 1988; Ren et al., 1999). Bell et al. (2020) found, in a nationally representative survey on health, functional, and nutritional status in the U.S. population, that Black Americans reported lower socioeconomic status and poorer subjective health.

Similarly, Boen (2016) suggests that higher SES is considered even more critical for Black American health where exposure to traumatic experiences, discrimination, and poverty in early life exists. Schoenbaum and Waidmann (1997), on the contrary, explored possible differences in levels of socioeconomic characteristics and their effects on the health status of Blacks and Whites. They concluded that Blacks were more likely to be disadvantaged concerning their SES (e.g., having less household income, owning a place of residents). These differences in SES may negatively influence their self-rated health (Schoenbaum and Waidmann, 1997). Moreover, Williams et al. (2016) found that the burden of racism, discrimination, and segregation was significant for all measures of SES (i.e., income, education, and occupation). This linkage between ethnic identity and poorer SES exposes minorities to many psychosocial stressors (Williams et al., 2016) and, simultaneously, limits access to healthcare resources (Nikoi and Odimegwu, 2013).

Therefore, this complex linkage between racial identity and SES could potentially explain how SES associates with quality of life, especially in a multiracial setting. A study from Jelsma and Ferguson revealed the impact of income and unemployment on the health-related quality of life (HRQoL) of South Africans. They explored possible preditors on HRQoL of residents of a socially and ethnically diverse suburb of Cape Town, South Africa. Their results show that belonging to a racial group, religious persuasion, and gender shows no direct influence on self-reported QoL. However, income and unemployment vary by race and significantly predict QoL (Jelsma and Ferguson, 2004). This finding stresses the need to explore the complex triangulation between QoL, SES, and racial identity in a racially diverse setting.

South Africa as a multiracial country (Adebanwi, 2017) allows for a thorough exploration of how racial identity moderates the association between SES and quality of life. The diverse racial and cultural identities in South Africa have historically influenced the structural and systemic designs of social, economic, and political spheres (Amoo et al., 2019). This is partly attributed to the racial segregation in apartheid South Africa. Understanding this association may provide essential insights into the mechanisms generating socioeconomic inequalities and their implication on life outcomes.

**Figure 1:**
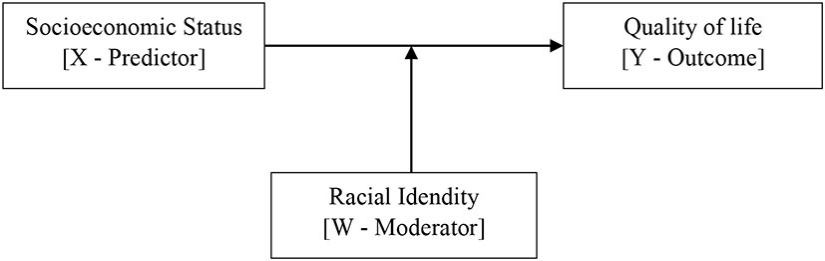
Conceptual model for racial identity as a moderator of the association between SES, QoL.

The current study aims to explore racial identity as a risk or protective factor that moderates the association between SES and QoL. To achieve this, the following objectives considering different constellations (see Figure 1) are set:

1. To explore racial differences in reported QoL scores,
2. To examine the association between SES and QoL for the total sample and racial groups,
3. To investigate racial identity as a moderator of the association between SES and participants' QoL outcomes.

## Method

### Study design and sample characteristics

Quantitative data on quality of life and socioeconomic and demographic characteristics were collected in a cross-sectional survey across the 9 provinces of South Africa. This was to ensure individuals from racial groups clustered in certain provinces had the chance to participate in the survey. A total of 1,062 South Africans completed an online questionnaire between January 2021 and September 2021. The questionnaire was administered online in the English language *via* the LimeSurvey Platform. A survey link and QR-code were generated and shared with potential participants. Cases with extensive missing data (more than 30% missing data) (*n* = 13) were removed from the dataset. Data from 1,049 were included in this analysis.

Participants were recruited using snowball sampling techniques (Goodman, 1961). Recruitment information was shared on social media (e.g., Facebook, Twitter, WhatsApp), the project webpage (www.beliv-study.com), and *via* networks and personal contacts. Purposive sampling was used to ensure the participants represented a variety of demographic and socioeconomic categories.

The average age of the participants is 26.36 years (SD = 7.14, range = 18–45). As shown in Table 1, about 60% are female. The frequency distribution of participants' racial identity shows about 78.6% identified as Black Africans, 11.3% as colored, 7.7% as Whites, and 2.4% as Indian South Africans.

**Table 1:** Sample characteristics (*n* = 1,049).

		***n***	**%**
Gender	Female	625	59.6
	Male	375	35.7
	Others	49	4.7
Racial identity	Black South African	824	78.6
	Colored South African	119	11.3
	White South African	81	7.7
	Indian South African	25	2.4
Educational attainment	None	28	2.7
	Primary	46	4.4
	Some secondary, excluding matric	175	16.7
	Matric or equivalent	427	40.7
	Tertiary education	340	32.4
	Doctorate/postdoctoral lecturing qualification	33	3.1
Total annual household income	Poor	691	65.9
	Low emerging middle class	71	6.8
	Emerging middle class	153	14.6
	Realized middle class	109	10.4
	Upper middle class	25	2.4
	Emerging affluent	-	-
Socioeconomic status	Very low	329	31.4
	Low	489	46.6
	Moderate	203	19.4
	High	28	2.7
	Very high	-	-

### Measures

#### Outcome–quality of life

Participants' subjective QoL was measured using the EUROHIS-QOL 8-item Index (Schmidt et al., 2006). This self-report questionnaire was derived from the WHO quality of life assessment (WHOQOL-100 and WHOQOL-BREF instruments) (The WHOQOL Group, 1998) and provides an aggregate subjective evaluation of life quality (Schmidt et al., 2006). It includes eight items representing the quality of life's physical, psychological, social, and environmental domains. The eight items were scored on a 5-point Likert scale ranging from “not at all” to “completely.” The overall quality of life score was computed as the eight items' aggregate scores ranging from 8 to 40, with higher scores indicating better QoL. The questionnaire presented good reliability in the current sample with a Cronbach's alpha value of 0.69.

#### Predictor–socioeconomic status

The SES was aggregated using the revised Socioeconomic Status Index of Lampert et al. (2013). Participants' household income, educational level, and occupation ranking were summed up to generate an SES score. The total score ranged from 3 to 18, with a higher score suggesting better SES. Scores from 3 to 6 were categorized as “very low,” 7–9 as “low,” 10–12 as “moderate,” 13–15 as “high,” and 16–18 as “very high.”

Household income was calculated by the family's approximate annual household income before taxes and other deductions (Maphupha, 2018). Income was measured with the South African currency Rand (R). Participants with annual income below R 54,344 were coded as “poor.” Participants with income between R 54,345 and R 151,727 as “low-emerging middle class,” R 151,728 to R 363,930 were coded as “emerging middle class,” R 363,931 to R 631,120 as “realized middle class,” R 631,121 to R 863,906 as “upper middle class,” and R 863,906 to R 1,329,844 as “emerging affluent” (Maphupha, 2018).

Education was assessed as the highest educational level, with options ranging from none to doctorate/postdoctorate (Brauns et al., 2003). Furthermore, participants were asked to rank how well their educational attainment matches their current occupation to measure their occupational level. In total, four options were provided, ranging from “I am not exercising an occupation at present” to “I am occupied above my qualification level.”

#### Moderator–racial identity

Following the South African government racial classification (Khalfani and Zuberi, 2001), participants' racial identity was measured by asking participants to select the racial group they most identify with. The participants were required to choose one of five options. These are Black African, Colored/mixed race, White, Indian South African, or others. Racial identity was dummy coded (Black African racial identity was coded as yes or no, the same for Colored, Indian, and White) for the regression analysis.

### Data analysis

**Figure 2:**
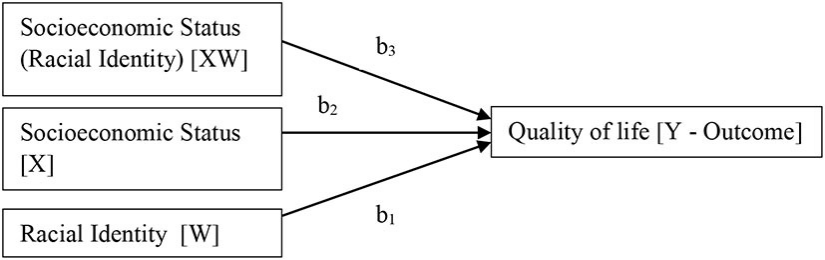
Statistical model—racial identity as a moderator of the association between socioeconomic status and quality of life.

Descriptive statistics were computed for sociodemographics, socioeconomic status, and quality of life. The ANOVA test was used to evaluate racial differences in quality of life. A correlation matrix exploring the bivariate associations between QoL and SES was computed. Correlation coefficients were interpreted as small (*r* = 0.10), medium (*r* = 0.30), or large (*r* = 0.50) Cohen, 2013). The data were checked for linear regression assumptions (outliner, linearity, zero-variance, autocorrelation, and homoscedasticity). Bootstrapping based on 1,000 samples was carried out to validate the results if assumptions were violated. Multiple linear regression models were calculated to assess the predictive effect of socioeconomic status on aggregate quality of life. A moderator analysis using the SPSS PROCESS macro version 4.0 was conducted to determine whether the relationship between socioeconomic status and quality of life is moderated by racial identity (see Figure 2).

Effect sizes and *p*-values were reported for the regression model. The overall fit of the models was evaluated by adjusted *R*^2^ statistics (Nagelkerke, 1991); *R*-change and *F*-test determined the significance of changes in model fit. To interpret the regression coefficients (β) of the regression models, we used guidelines by Cohen (2013): β = 0.1 indicated a small, β = 0.3 a medium, and β = 0.5 a large effect. The significance level was determined as *p* < 0.05 for all the analyses. Analyses were computed using IBM SPSS Version 26.

## Results

### Quality of life

**Figure 3:**
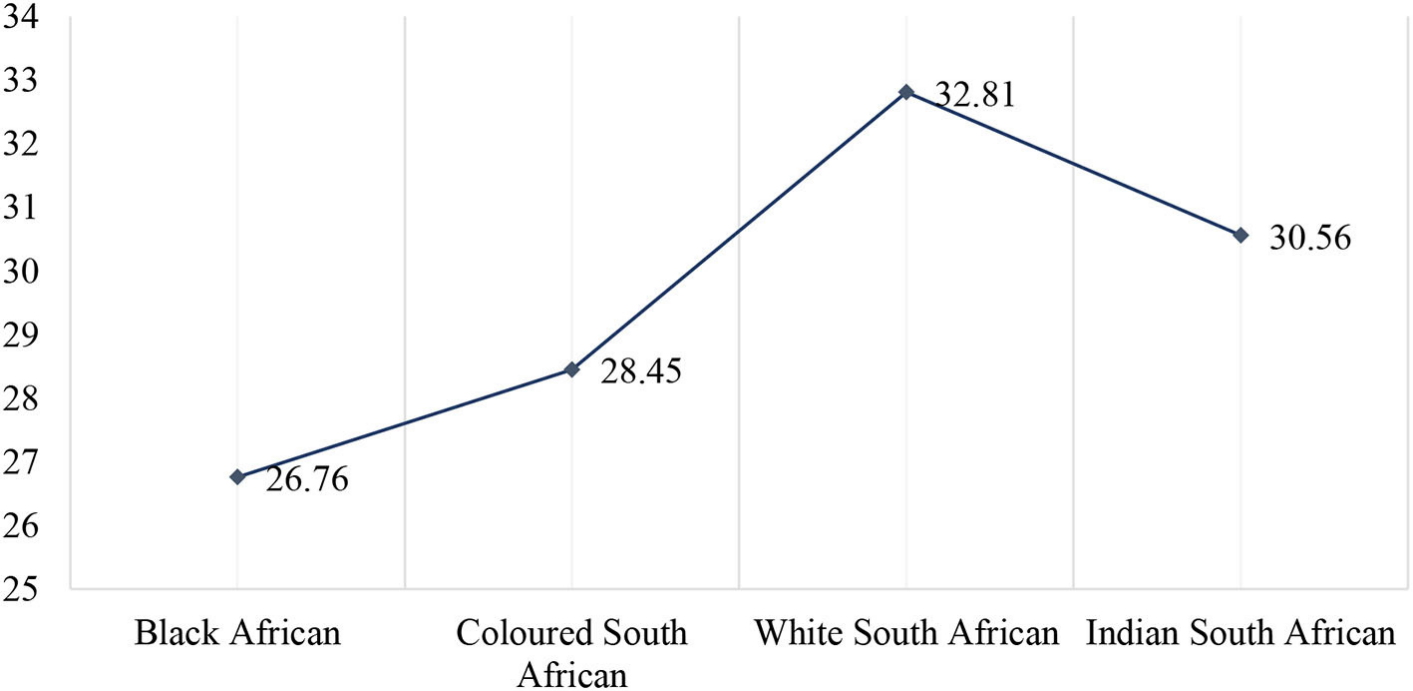
Quality of life scores by racial identity.

The sample QoL scores ranged between 11 and 40 for the current sample. Descriptive analysis returned a mean score of 27.51 (SD = 5.07) for the total sample. As shown in Table 2 above, six paired samples *t*-tests using the *post-hoc* Scheffé test indicated significant differences in the mean quality of life score by racial identity: Black Africans reported ranging score from 11 to 40 and an average of 26.76 scores (SD = 4.83), significantly lower than Colored/mixed-race participants (Min = 14, Max = 36, M = 28.45, SD = 5.57), Indians (Min = 18, Max = 35, M = 30.56, SD = 3.45), and White South Africans (Min = 23, Max = 38, M = 32.81, SD = 2.98) average quality of life score (see Figure 3).

**Table 2:** Quality of life score multiple comparisons by racial identity: Scheffé *post-hoc* criterion.

**(I) Racial identity**	**(J) Racial identity**	**Mean difference (I-J)**	**Std. error**	**Sig**.
Black African	Colored	−1.69286^*^	0.46836	0.005
	White	−6.05389^*^	0.55613	0.000
	Indian	−3.79908^*^	0.96956	0.002
Colored/Mixed race	White	−4.36103^*^	0.68795	0.000
	Indian	−2.10622	1.05074	0.260
White	Indian	2.25481	1.09269	0.236

* The mean difference is significant at the 0.05 level.

95% Confidence interval.

### Socioeconomic status

Descriptive analysis of the aggregate socioeconomic status returned scores between 3 and 15 for the current sample. An average score of 7.98 (SD = 2.15) was computed for the total sample. Categorized data suggested that about 78% of the participants reported “low” or “very low” socioeconomic status (see Table 1).

A one-way ANOVA showed that the effect of racial identity was significant, *F* (3, 1045) = 35.98, *p* < 0.001 for socioeconomic status. *Post-hoc* analyses using the Scheffé *post-hoc* criterion for significance indicated that the average socioeconomic score was significantly lower in Black (M = 7.81, SD = 1.99) than in White South Africans (M = 10.22, SD = 2.66), *p* < 0.001 and not significantly different from Colored and Indian South Africans. Similarly, the mean socioeconomic status score for Colored participants (M = 7.55, SD = 1,99) was significantly different from White Africans' socioeconomic status (*p* < 0.001) but not different from Black Africans and Indian South Africans' socioeconomic status scores. AT last, the average socioeconomic status score for Indian South African participants (M = 8.16, SD = 1.89) was significantly different from White (*p* < 0.001) but not from Blacks and Colored (see Table 3).

**Table 3:** Socioeconomic status score multiple comparisons by racial identity: Scheffé *post-hoc* criterion.

**(I) Racial identity**	**(J) Racial identity**	**Mean difference (I-J)**	**Std. error**	**Sig**.
Black African	Colored	0.25606	0.20093	0.654
	White	−2.41154^*^	0.23859	0.000
	Indian	−0.34932	0.41597	0.872
Colored/Mixed race	White	−2.66760^*^	0.29515	0.000
	Indian	−0.60538	0.45079	0.614
White	Indian	2.06222^*^	0.46879	0.000

* The mean difference is significant at the 0.05 level.

95% Confidence interval.

### Bivariate analysis

Pearson product moment correlation coefficient matrix was computed to examine the association between the quality of life and socioeconomic status. The results show that participants quality of life score moderately correlates with socioeconomic score, *r* (1,047) = 0.199, *p* < 0.01 (two-tailed).

### Regression

**Figure 4:**
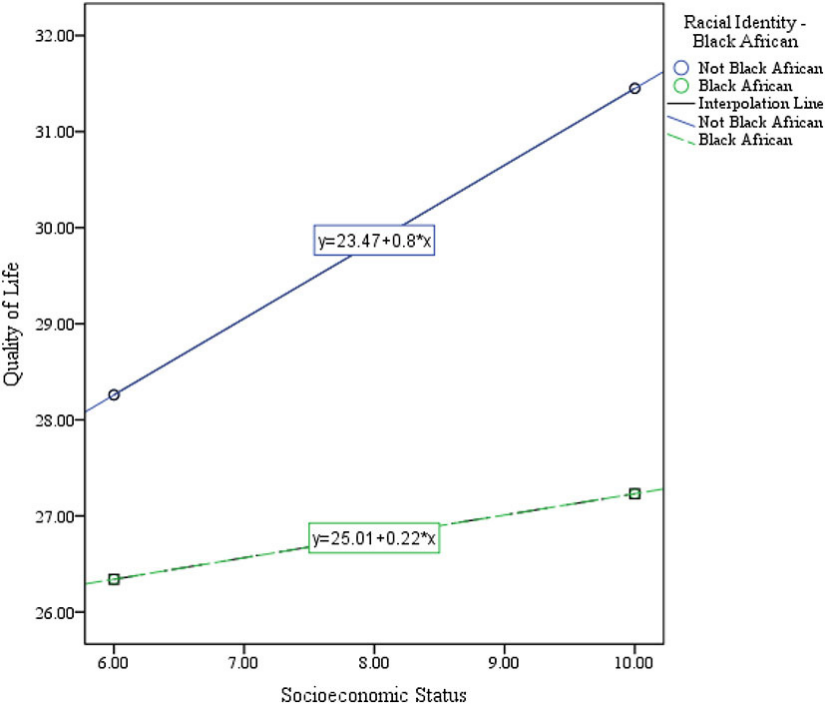
Black African racial identity as a moderator of the association between SES and QoL.

The regression model (Model 1, see Table 4) shows a statistically significant positive association between QoL, SES, and racial identity *F* (4, 1,044) = 35.18, *p* < 0.001, R^2^ = 0.12 for participants with Black African racial identity. The model further suggest a 0.80 unit increase in quality of life score for every unit increase in SES, *b* = 0.80, *t* (1,044) = 6.28, *p* < 0.001. The dummy coded variable racial identity for Black African was not significant in the model β = 1.53, *t* (1,044) = 1.18, *p* = 0.24. However, the interaction effect between racial identity, and SES shows significant association with QoL score *b* = −0.58, *t* (1,044) = −3.83, *p* < 0.001. Furthermore, the moderating effect of racial identity suggests that a point increase in SES for Black African participants correspond to 0.22 unit increase in quality of life, *b* = 0.22, *t* (1044) = 2.63, *p* = 0.01 (see Figure 4).

**Table 4:** Socioeconomic status and quality of life.

	**Model 1**			**Model 2**			**Model 3**			**Model 4**		
	**Black African**			**Colored South African**			**White South African**			**Indian South African**		
	**β**	**t**	***p***	**β**	**t**	***p***	**β**	**t**	***p***	**β**	**t**	***p***
***Constant***	24.22	20.42	0.00	23.60	37.76	0.00	25.16	40.36	0.00	23.66	39.91	0.00
Socioeconomic status	**0.80**	**6.28**	**0.00**	**0.47**	**6.29**	0.00	**0.25**	**3.16**	**0.00**	**0.47**	**6.59**	**0.00**
Racial identity	1.53	1.18	0.24	0.47	0.25	0.80	2.49	1.12	0.26	6.15	1.36	0.17
***Interaction*** SES*Racial identity	**−0.58**	**−3.83**	**0.00**	0.11	0.45	0.66	0.26	1.21	0.23	**–**0.38	**–**0.71	0.48
Adjusted R^2^	0.12			0.05			0.11			0.05		
F (df1, df2)	F (4, 1,044) = 35.18			F (3, 1,045) = 16.92			F (3, 1,045) = 41.17			F (3, 1,045) = 17.73		
*p*-value	*p* <0.001			*p* <0.001			*p* <0.001			*p* <0.001		

Model 1: Moderating effect of Black African racial identity on the association between socioeconomic status and quality of life, and predictive effect of quality of life.

Model 2: Moderating effect of Colored South African racial identity on the association between socioeconomic status and quality of life, and predictive effect of quality of life.

Model 3: Moderating effect of White South African racial identity on the association between socioeconomic status and quality of life, and predictive effect of quality of life.

Model 4: Moderating effect of Indian South African racial identity on the association between socioeconomic status and quality of life, and predictive effect of quality of life.

Highlights significant values.

Model 2 also shows a significant positive association between quality of life, socioeconomic status, and racial identity *F* (3, 1,045) = 16.92, *p* < 0.001, R^2^ = 0.05 for participants with Colored South African racial identity. The model further suggests a 0.47 unit increase in QoL score for every unit increase in SES, β = 0.47, *t* (1,045) = 6.29, *p* < 0.001. The dummy coded variable racial identity for Colored Africans, and the interaction variable was not statistically significant in the model. Similarly, Models 3 and 4, accessing the moderating effect of racial identity for White and Indian South African participants, suggest racial identity does not moderate the association between quality of life, and socioeconomic status for both racial groups.

## Discussion

Racial identity is an important concept that indicates a broad knowledge of how people or individuals are defined based on race, ethnicity, and cultural affinity (Neblett et al., 2016). This study aimed to explore racial differences in QoL outcomes and to investigate the moderating effect of race on the association between SES and QoL across the nine provinces of South Africa.

This study confirms that Black Africans scored lowest in the QoL compared with their counterparts, Colored, Indians, and White South Africans. This finding collaborates with findings from the South African quality of life trends (Møller, 2013). In alignment with results from the current study, Møller (2013) concluded that the subjective wellbeing of South Africans—measured by indicators of life satisfaction, happiness, and perceptions of progress—varies significantly with race (Black, Colored, Indian, and White). From 1983 to 2010, Black Africans reported the lowest satisfaction with life-as-a-whole and global happiness in all waves (except waves 1994 and 1997). While the results from the last wave (2010) suggested a worsening performance for Black Africans over the years, White South Africans reported the highest satisfaction with current and future life (Møller, 2013).

Further results on the determinant of QoL confirm SES as a predictor of South Africans QoL. SES shows a significant association with the quality of life of all racial groups. However, results for Black Africans suggest that one of the most common explanations of the poor QoL outcome for Black Africans can be seen in their overall disadvantaged situation resulting from South Africa's history and its related racism (Gaibie and Davids, 2009). The racial segregation in apartheid South Africa led to an inequitable distribution of resources in the form of income, job opportunities, and access to wealth between Black Africans and Whites. This inequality between different ethnic groups is arguably attributed to their SES and the significant differences in QoL outcome. Therefore, the current study results agree with other studies that argued that these diverge in SES might still predict South Africans QoL post-apartheid (Boen, 2016; Bell et al., 2020).

Further analyses explore racial identity as a moderator of the established association between SES and QoL. While the result confirms Black African racial identity as a significant moderator of the association between SES and QoL, other racial identities show no significant moderating effect on the association between SES and QoL. This result further emphasizes the socioeconomic disadvantage directly related to black racial identity and its strong implication for life outcomes (Georgopoulou et al., 2011; Keyvanara et al., 2015). Although all historically marginalized groups (Blacks, Colored, and Indians) experienced discrimination in the apartheid era, the racial classifications that put Whites at the top and Black Africans at the bottom (Williams et al., 2008) arguably tailored a unique disadvantaged experience for Black Africans. This experience of discrimination is related to psychological stress. It may also explain the poor SES and its association with racial identity, making Black African life outcomes more susceptible to social and economic change (Yip, 2018). Black African participants in this study reported the lowest SES and QoL score. This performance can be connected with the experience of discrimination and accumulated inequalities. Furthermore, among the ethnic identified groups, the Black Africans have shown a high level of belonging and social ties (HSRC, 2021). Thus, equal racial classification of Black Africans is evident in the trajectory of socioeconomic status and the QoL of the group.

### Limitations of the study

Despite the empirical contribution of the present study, it is essential to note that the adapted cross-sectional design limits the interpretation and generalizability of the results. It remains unclear how QoL and racial identity may change or not change after some decades post-apartheid. While participants from all the four racial groups were invited to participate in the online study, the adopted sampling technique might have limited the chances of participation and caused selection bias. In this study, Indian participants were underrepresented. However, a similar trend was reported in a survey by Gaibie and Davids, where 76% of the participants were Black Africans, and only 3% of 3,321 participants were Indian/Asian. This is explained by their minority stand in the population and the challenge in accessing this group (Gaibie and Davids, 2009).

## Conclusion

The study provides information about the QoL, SES, and racial identity's moderating effect on the association between Qol and SES. The results emphasize the implication of Black African racial identity for life outcomes and show that the negative impact of segregation remains decades post-apartheid. These results provide policymakers with initiatives to prevent and remove social and economic disadvantages to improve health. Furthermore, it provides a more straightforward path to reducing inequality and thereby facilitating sustainable development goals (SDGs). Besides policies to support the social and economic situation of Black Africans, they should be actively involved in health-promoting programs aiming to improve their QoL.

Future research should differentiate more strongly between contexts of acculturation and social determinants of health (e.g., discrimination, racial, ethnic support) for their policy implications. Similarly, the results reported should be investigated in minority groups underrepresented in this study (e.g., Indian). Other features, such as social situation and health behavior, should be considered. Qualitative research methods, such as focus groups or narrative interviews, are needed to understand different (individual) health concepts and their association with SES and racial identity.

## Author's note

The racial categories, i.e., Black, Colored, White, and Indian, adopted in this study are based on the South African official racial classification as reported by Statistics South Africa (http://www.statssa.gov.za/publications/P0318/P03182019.pdf Accessed on 19.05.2022).

## Data availability statement

The raw data supporting the conclusions of this article will be made available by the authors, without undue reservation.

## Ethics statement

The studies involving human participants were reviewed and approved by Basic and Social Sciences Research Ethics Committee (BaSSREC) of the North-West University: NWU-00617-21-A7. The patients/participants provided their written informed consent to participate in this study.

## Author contributions

AA conceptualized the study, collected and analyzed data, and contributed to manuscript writing. JB and EI project consultation and contributed to manuscript writing. TA contributed to manuscript writing. All authors contributed to the article and approved the submitted version.

## Funding

This study was conducted as part of a Feodor Lynen Research Fellowship, funded by the Alexander von Humboldt Foundation awarded to AA.

## Conflict of interest

The authors declare that the research was conducted in the absence of any commercial or financial relationships that could be construed as a potential conflict of interest.

## Publisher's note

All claims expressed in this article are solely those of the authors and do not necessarily represent those of their affiliated organizations, or those of the publisher, the editors and the reviewers. Any product that may be evaluated in this article, or claim that may be made by its manufacturer, is not guaranteed or endorsed by the publisher.
